# A combined meta-barcoding and shotgun metagenomic analysis of spontaneous wine fermentation

**DOI:** 10.1093/gigascience/gix040

**Published:** 2017-06-08

**Authors:** Peter R. Sternes, Danna Lee, Dariusz R. Kutyna, Anthony R. Borneman

**Affiliations:** 1 The Australian Wine Research Institute, PO Box 197, Glen Osmond, South Australia, 5064; 2 Department of Genetics and Evolution, University of Adelaide, South Australia, 5000 Present address; 3 Institute of Health and Biomedical Innovation, Queensland University of Technology, Wooloongabba, Queensland, Australia; 4 Department of Cell and Molecular Biology, Uppsala University, Uppsala, Sweden

**Keywords:** wine, metagenomics, phylotyping, yeast diversity

## Abstract

Wine is a complex beverage, comprising hundreds of metabolites produced through the action of yeasts and bacteria in fermenting grape must. Commercially, there is now a growing trend away from using wine yeast (*Saccharomyces*) starter cultures, toward the historic practice of uninoculated or “wild” fermentation, where the yeasts and bacteria associated with the grapes and/or winery perform the fermentation. It is the varied metabolic contributions of these numerous non-*Saccharomyces* species that are thought to impart complexity and desirable taste and aroma attributes to wild ferments in comparison to their inoculated counterparts. To map the microflora of spontaneous fermentation, metagenomic techniques were employed to characterize and monitor the progression of fungal species in 5 different wild fermentations. Both amplicon-based ribosomal DNA internal transcribed spacer (ITS) phylotyping and shotgun metagenomics were used to assess community structure across different stages of fermentation. While providing a sensitive and highly accurate means of characterizing the wine microbiome, the shotgun metagenomic data also uncovered a significant overabundance bias in the ITS phylotyping abundance estimations for the common non-*Saccharomyces* wine yeast genus *Metschnikowia*. By identifying biases such as that observed for *Metschnikowia*, abundance measurements from future ITS phylotyping datasets can be corrected to provide more accurate species representation. Ultimately, as more shotgun metagenomic and single-strain *de novo* assemblies for key wine species become available, the accuracy of both ITS-amplicon and shotgun studies will greatly increase, providing a powerful methodology for deciphering the influence of the microbial community on the wine flavor and aroma.

## Background

Wine is a complex beverage, comprising thousands of metabolites that are produced through the action of yeasts and bacteria in fermenting grape must. When grapes are crushed and allowed to ferment naturally, a complex microbial succession of yeasts and bacteria is generally observed. In the very early stages of fermentation, aerobic and apiculate yeasts and yeast-like fungi from genera such as *Aureobasidium*, *Rhodotorula*, *Pichia*, *Candida*, *Hanseniaspora* and *Metschnikowia*, which reside on the surface of intact grape berries or winery equipment, represent the majority of the microbiota [1].

However, most of these species, especially the aerobic yeasts, succumb early in the succession of the fermentation in response to falling oxygen levels and increasing ethanol. Mildly fermentative yeasts, such as *Hanseniaspora uvarum*, *Candida stellata*, *Metschnikowia pulcherrima, Torulaspora delbrueckii*, and *Lachancea thermotolerans*, can proliferate and survive well into the fermentation, but fall in numbers as ethanol levels increase, although it has been reported that *C. stellata* can survive up to 12% ethanol and complete fermentation [2–5].

Despite the vastly higher numbers of non-*Saccharomyces* yeasts early in the fermentation process, the major wine yeast, *Saccharomyces cerevisiae*, is responsible for the bulk of the ethanolic fermentation. However, *S. cerevisiae* is not readily isolated from intact grape berries and is therefore generally found in very low numbers at the start of fermentation [6, 7]. Regardless, due to its higher fermentative ability, growth rate, and tolerance to ethanol, *S. cerevisiae* supplants the various non-*Saccharomcyes* yeasts, becoming the dominant species from mid-fermentation such that an almost monoculture of this 1 species is established by the end of fermentation.

While traditional microbiological techniques have provided important insights into the microbial succession that occurs in spontaneous ferments, both the breadth of ferments investigated and the depth at which individual species contributions could be resolved have been limited. Recent advances in culture-independent methods for species analysis, such as amplicon-based phylotyping (also known as meta-barcoding) and metagenomics provide a high-throughput means to analyze large numbers of microbiological samples at great depth [8]. Accordingly, these techniques are now being adapted for the study of wine fermentation, and several amplicon-based studies have been conducted to investigate vineyard and wine microbiomes [9–14].

However, despite these previous amplicon studies, there are still concerns regarding biases that may be inherent in the process due to uneven polymerase chain reaction (PCR) amplification or unequal copy number of the ribosomal repeat [9, 15]. To address some of these limitations, metagenomic techniques are being used to determine species abundance from shotgun sequencing of mixed samples. These techniques generally rely on read mapping, either to collections of curated marker genes or whole genomes, making them reliant on reference sequences that are available [16]. As yet, shotgun metagenomics has not been applied to the study of wine fermentation or to assess the accuracy of amplicon-based abundance estimates of wine fermentation.

Fungal-specific internal transcribed spacer (ITS) phylotyping was performed over 4 key fermentation stages in 5 independent commercial Chardonnay juice fermentations in triplicate. Full shotgun metagenomic sequencing was also performed for 20 of these samples. Comparison of the ITS phylotyping and shotgun data uncovered a major amplicon bias that existed for the genus *Metschnikowia*, providing the means to normalize other ITS datasets in which this species is abundant.

## Data Description

To map the microflora of spontaneous fermentation, both amplicon-based ITS phylotyping (meta-barcoding) and shotgun metagenomics were used to assess community structure, while comparing the biases inherent in PCR-based phylotyping relative to the unbiased shotgun metagenomic sequencing. These data provide key insights into the species participating in wild wine fermentation, while also highlighting the at least 1 significant bias in the ITS analysis of wine yeasts. All sequencing data, including ITS barcoding and shotgun metagenomic sequencing, have been deposited in Genbank under the Bioproject accession number PRJNA305659. The datasets supporting the results of this article are available in the GigaDB repository [17].

## Analyses and Discussion

### Analysis of microbial communities in wild ferments

To study the reproducibility and applicability of performing metagenomics analyses of laboratory-scale uninoculated ferments, 5 Chardonnay grape musts (Y1, Y2, Y3, T1, and T2), which were each destined to undergo winery-scale uninoculated fermentation (sourced from 2 different wineries), were fermented at laboratory scale in triplicate (Table 1). Fermenting musts were tracked for sugar consumption via refractometry, with samples taken for analysis at 4 key time points: D0, just after crush (11–12 Baumé [Bé]); D1, the onset of fermentation (10–11 Bé, ∼90% residual sugar); D2, mid-ferment (5–6 Bé, ∼50% residual sugar); and D3, nearing the end of ferment (<3 Bé, <25% residual sugar). All ferments proceeded to dryness (0 Bé, <5 g/l residual sugar), with sample Y1 being the fastest (12 days) and sample T1 taking the longest time (27 days) to complete fermentation.

**Table 1:** Fermentation samples used in this study

	Grape			ITS	Shotgun
Sample	variety	Vineyard; winery location	Stage of ferment	samples^a^	samples^b^
T1	Chardonnay	Adelaide Hills, SA; Barossa Valley, SA	At crush (100% sugar)	T1 D0	
			90% residual sugar	T1 D1	T1 D1
			50% residual sugar	T1 D2	
			10–20% residual sugar	T1 D3	
T2	Chardonnay	Adelaide Hills, SA; Barossa Valley, SA	At crush	T2 D0	
			90% sugar	T2 D1	T2 D1
			50% sugar	T2 D2	
			10–20% sugar	T2 D3	T2 D3
Y1	Chardonnay	Eden Valley, SA; Barossa Valley, SA	At crush	Y1 D0	
			90% sugar	Y1 D1	Y1 D1
			50% sugar	Y1 D2	
			10–20% sugar	Y1 D3	
Y2	Chardonnay	Eden Valley, SA; Barossa Valley, SA	At crush	Y2 D0	Y2 D1
			90% sugar	Y2 D1	
			50% sugar	Y2 D2	
			10–20% sugar	Y2 D3	Y2 D3
Y3	Chardonnay	Adelaide Hills, SA; Barossa Valley, SA	At crush	Y3 D0	
			90% sugar	Y3 D1	Y3 D1
			50% sugar	Y3 D2	
			10–20% sugar	Y3 D3	Y3 D3

^a^Sequencing was performed on biological triplicates (samples A, B, and C).

^b^Sequencing was performed on biological duplicates (samples A and B).

### Species abundance estimation via ITS-amplicon analysis

A total of 66 samples were analyzed comprising 6 control populations (2 different mock communities in triplicate) and 60 laboratory-scale fermentations (4 stages during ferment in triplicate for 5 different musts) (Table S1). DNA was isolated from the pelleted fraction of each must sample, with a 2-step PCR performed using sequences designed to amplify the fungal ITS region [9], while adding experiment-specific inline barcodes and appropriate adaptors for sequencing on the Illumina sequencing platform (Fig. S1). Following sequencing and barcode and adaptor trimming, 8.8 million reads were assigned across the samples (Table S1), with an average of over 100 000 reads per sample.

To consistently describe and compare the number of operational taxonomic units (OTUs) across the samples, all 8.8 million reads were first analyzed as a large single batch. Dereplication [18], OTU clustering [19], and taxonomic assignment [20] of this combined dataset resulted in the production of a single OTU table that encompassed all the OTUs from across all 66 samples. Abundance measurements of each individual dereplicated OTU from each sample were then mapped to this combined data table to derive the contribution of each experiment to the collective dataset (Table S2).

### Mock control populations

Given previous concerns regarding the accuracy of ITS-amplicon profiling [9], 2 different mock control populations were assembled, in triplicate, from known numbers of cells obtained from individual cultures of 7 common wine-associated yeasts, representing 6 different species and 5 different genera (Table 2). By comparing the results of the ITS-amplicon profiling of these samples with those expected from estimated numbers of input cells, nearly all species estimates were within 2-fold of their expected value, despite cell concentrations differing across 5 orders of magnitude (Table 2). However, the results for *Metschnikowia* appeared to be reproducibly overestimated in both control populations (18.6- and 10.5-fold), indicating that this species may display significant amplicon bias for the ITS region relative to the other samples used in the control populations.

**Table 2:** Composition of control populations and comparison of phylotyping and shotgun metagenomics abundance measurements

		Control	Total	ITS abundance	Shotgun	Control	Total	ITS abundance	Shotgun
Strain	Species	mix 1	OTUs	(ratio)^a^	abundance (ratio)^a^	mix 2	OTUs	(ratio)^a^	abundance (ratio)^a^
AWRI796	*Saccharomyces cerevisiae*	1 × 10^6^	3	1 × 10^6^ (1)^b^	1 × 10^6^ (1)^b^	1 × 10^8^	11	2 × 10^8^ (1)^b^	2 × 10^8^ (1)^b^
AWRI1498	*Saccharomyces cerevisiae*	1 × 10^4^				1 × 10^8^			
AWRI1149	*Metschnikowia pulcherrima*	1 × 10^4^	7	1.9 × 10^5^ (18.6)	8.8 × 10^3^ (0.9)	1 × 10^6^	7	1.1 × 10^7^ (10.5)	6.5 × 10^5^ (0.7)
AWRI1152	*Torulaspora delbrueckii*	1 × 10^6^	1	7.3 × 10^5^ (0.7)	4.8 × 10^5^ (0.5)	1 × 10^5^	1	6.6 × 10^4^ (0.7)	4.7 × 10^4^ (0.5)
AWRI1157	*Debaryomyces hansenii*	1 × 10^7^	1	8.4 × 10^6^ (0.8)	2.9 × 10^6^ (0.3)	1 × 10^3^	1	2.4 × 10^3^ (2.4)	0.0
AWRI1176	*Saccharomyces uvarum*	1 × 10^3^	1	5.7 × 10^2^ (0.6)	1.8 × 10^3^ (1.8)	1 × 10^5^	3	7.9 × 10^4^ (0.8)	1.1 × 10^5^ (1.1)
AWRI1274	*Haneniaspora uvarum*	1 × 10^8^	4	1.1 × 10^8^ (1.1)	1.3 × 10^8^ (1.3)	1 × 10^4^	2	6.1 × 10^4^ (6.1)	4.4 × 10^4^ (4.4)

^a^The ratios are presented as the observed abundance/expected abundance (the total number of cells added to the control mix).

^b^All data were internally normalized for comparison by setting the observed abundance of *S. cerevisiae* to a final ratio of 1.

### Laboratory ferments

**Figure 1:**
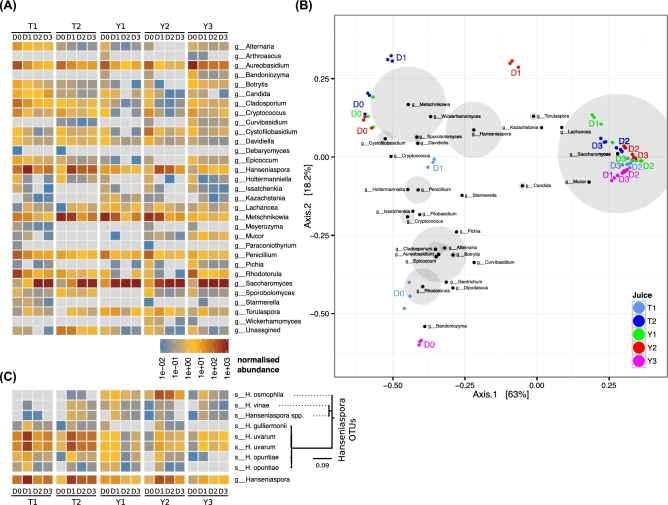
ITS amplicon abundance of uninoculated ferments. **(A)** Laboratory-scale ferments analyzing 4 fermentation time points in 5 different musts in triplicate. ITS sequences are grouped by genus and are colored-coded by their normalized abundance (reads per thousand reads). **(B)** Dissimilarity analysis of ITS-amplicon abundance. Triplicate samples from each time point were subjected to Bray-Curtis dissimilarity analysis. The PCA weightings of the top 30 genera are overlaid on the plot, with the size of the gray circles around each node proportional to the total abundance of each genus across all samples (no shading for nodes >5000 counts). **(C)** Species-level ITS assignment for the genus *Hanseniaspora*. The individual abundance measurements for the 8 OTUs that comprise the g__Hanseniaspora category are shown, grouped by phylogenetic distance. Results are color-coded according to normalized abundance (reads per thousand reads).

ITS-amplicon analysis of laboratory-scale wild ferments showed that there was a high degree of reproducibility between each of the 3 biological triplicates (r^2^ = 0.95 ± 0.02) (Fig. S2). All the fermentations displayed an expected microbiological succession, beginning with a diverse and variable collection of fungi that progressively resolved into a population that was dominated by the major wine yeast *S. cerevisiae* (Fig. 1A). Multidimensional scaling (principal coordinate analysis, using Bray-Curtis distance) of the ferments showed that while T2, Y1, an Y2 could be broadly classified as being dominated by *Metschnikowia* and *Hanseniaspora* at the D0 and D1 time points, the Y3 ferment was almost devoid of these genera, with the ferment characterized by high levels of *Aureobasidium* and *Rhodotorula*, primarily at D0 (Fig. 1B). T1, the slowest ferment, displayed a highly diverse D0 population of *Rhodotorula*, *Cladosporium*, and *Aureobasidium*, which progressed through a *Hanseniaspora*-dominated phase at D1 and finally to *S. cerevisiae* at D2/D3. These dominant species, and their progression during fermentation, are broadly similar to those found in previous studies of wine fermentation [12, 14].

The use of the fungal ITS marker also allowed for species-level assignment of many OTUs, and there were several genera for which more than 1 species was encountered. For example, the genus *Hanseniaspora* was represented by a total of 8 OTUs that could be grouped into at least 5 main species (by ITS sequence similarity; *H. uvarum*, *H. opunitae*, *H. osmophila*, *H. vineae*, and *H. guilliermondii*) (Fig. 1C), with 2 species, *H. uvarum* and *H. opuntiae*, having 2 distinct OTUs representing each species, but which displayed coordinated changes in abundance across both juice and time point. For these species, this argues that either there were multiple strains of each species present in the ferments (with slightly different ITS sequences) that were responding similarly or that multiple OTU sequences were being produced per species (either due to heterogeneous ITS repeats or PCR artefacts).

Interestingly, these 2 main categories of ferments that were observed (T2, Y1 and Y2, vs T1 and Y3) did not correlate with vineyard location and/or winery (Table 1). However, the overall difference in the location of the vineyards and wineries is relatively minor, with Eden Valley and the Adelaide Hills being geographically adjacent regions in South Australia. The driver of these differences in microbial starting populations and progressions therefore remains to be determined; however, undocumented factors such as vineyard management and/or microclimate may be involved [21–24].

### Shotgun metagenomics

While ITS-amplicon sequencing provided an in-depth analysis of variation across ferments, it has been widely accepted that the combination of ITS primer sequences, multiple rounds of PCR, and variation in the ITS repeat number can produce biases in the final abundance measurements [9, 15]. In addition, unless a second primer set is employed, bacterial species are not covered by this analysis. To explore these potential biases in more detail and to potentially provide strain-level information, shotgun metagenomics, in which total DNA is extracted and directly sequenced, was employed on a total of 20 of the samples analyzed by ITS-amplicon sequencing (Table 3).

**Table 3:** Shotgun metagenomic read alignment statistics

		Read alignment
Sample	Total reads	rate^a^ (%)
Control mix 1 replicate A	18 816 478	97.05
Control mix 1 replicate C	17 883 449	97.11
Control mix 2 replicate A	20 343 317	97.18
Control mix 1 replicate C	18 138 322	97.91
T1 D1 replicate A	20 027 063	88.15
T1 D1 replicate B	21 175 617	90.22
T2 D1 replicate A	18 173 778	90.12
T2 D1 replicate B	21 705 055	91.02
T2 D3 replicate A	21 282 134	97.12
T2 D3 replicate B	21 112 818	96.84
Y1 D1 replicate A	20 196 267	96.04
Y1 D1 replicate B	20 404 693	96.39
Y2 D1 replicate A	18 384 104	93.13
Y2 D1 replicate B	18 960 301	92.61
Y2 D3 replicate A	20 731 661	96.66
Y2 D3 replicate B	19 327 663	96.75
Y3 D1 replicate A	19 843 426	94.48
Y3 D1 replicate B	21 559 192	94.29
Y3 D3 replicate A	19 533 468	96.69
Y3 D3 replicate B	19 258 370	95.77

^a^Sequencing reads from each sample (pre-filtered to remove grapevine matches) were aligned against the wine reference consortium (Table S3).

Given that reference genome sequences exist for many wine-associated microbes, a mapping abundance strategy was used to analyze the shotgun data. A representative collection of reference genomes was therefore obtained from existing genomic resources for fungal and bacterial genera that were known or suspected of being wine associated (Table S3). However, attempts at aligning to a preliminary reference genome set for shotgun abundance estimation (see below) resulted in up to 15% of reads being unable to be aligned.

To determine if this was due to a lack of suitable reference genomes for key species that were represented in the shotgun data, all unaligned sequences were subsequently *de novo* assembled, with the resulting contigs partitioned according to their likely genus. Four genera were represented by at least 500 kb of sequence, although 3 of these *Rhodosporidium*, *Rhodotorula*, and *Microbotryum*, represent synonymous species and were combined and ascribed to a single major assembly product, *Rhodosporidium* (*n* = 1539, 17.7 Mb). As 3 other species of *Rhodosporidium* and *Rhodotorula* are present in the reference dataset, these contigs likely represent the genome of an additional species within this genus.

A fourth group of contigs (*n* = 76, 644 kb) was ascribed to *Aureobasidium spp.*, despite the presence of a reference sequence for *Aureobasidium pullulans*. However, in this instance, the strong correlation in abundance values obtained across the samples for these 2 different sequences point to these *de novo* contigs representing regions that are not conserved in the existing *A. pullulans* reference sequences (Fig. S3). These sequences were subsequently added to the reference set for use in the shotgun abundance estimation.

### Estimating species abundance using shotgun metagenomic sequencing

The final reference genome set comprised 851 Mb of DNA that represented a total of 51 species (45 eukaryotic, 6 prokaryotic) (Table S3). After filtering each sample for reads that matched the grapevine reference genome (<1% per sample), each filtered sample was aligned to the reference genome set. This approach resulted in most reads being able to be matched to the reference consortium, although this was highly sample dependent, with between 2% and 11% of reads not able to be aligned adequately to the reference set of sequences (Table 3). These unaligned sequences likely represent species for which an adequate reference genome was not present in the consortium, while being present at too low of an abundance to produce significant contigs during the *de novo* assembly of the unaligned pool.

In order to determine the potential taxonomic source of these unaligned reads, the marker-gene metagenomic classifier MetaPhlAn [25] was used to classify the remaining reads from this dataset (Table S4). This indicated 40% of the remaining reads to be of bacterial origin, 46% from *Ascomycetous* fungi and 13% viral. The most highly represented bacterial genera included *Acetobacter* (25% total bacterial reads), *Curtobacterium* (14%), and *Lactobacillus* (18%), which are all commonly associated with wine or grapes [12, 26]. Of the *Ascomycete spp.*, half the reads were predicted to be from *S. cerevisiae* and likely represent mitochondrial reads (the mitochondrion was excluded from the reference genome set due to its variable copy number), with another 30% predicted to derive from an unclassified member of the family *Debaryomycetaceae*.

**Figure 2:**
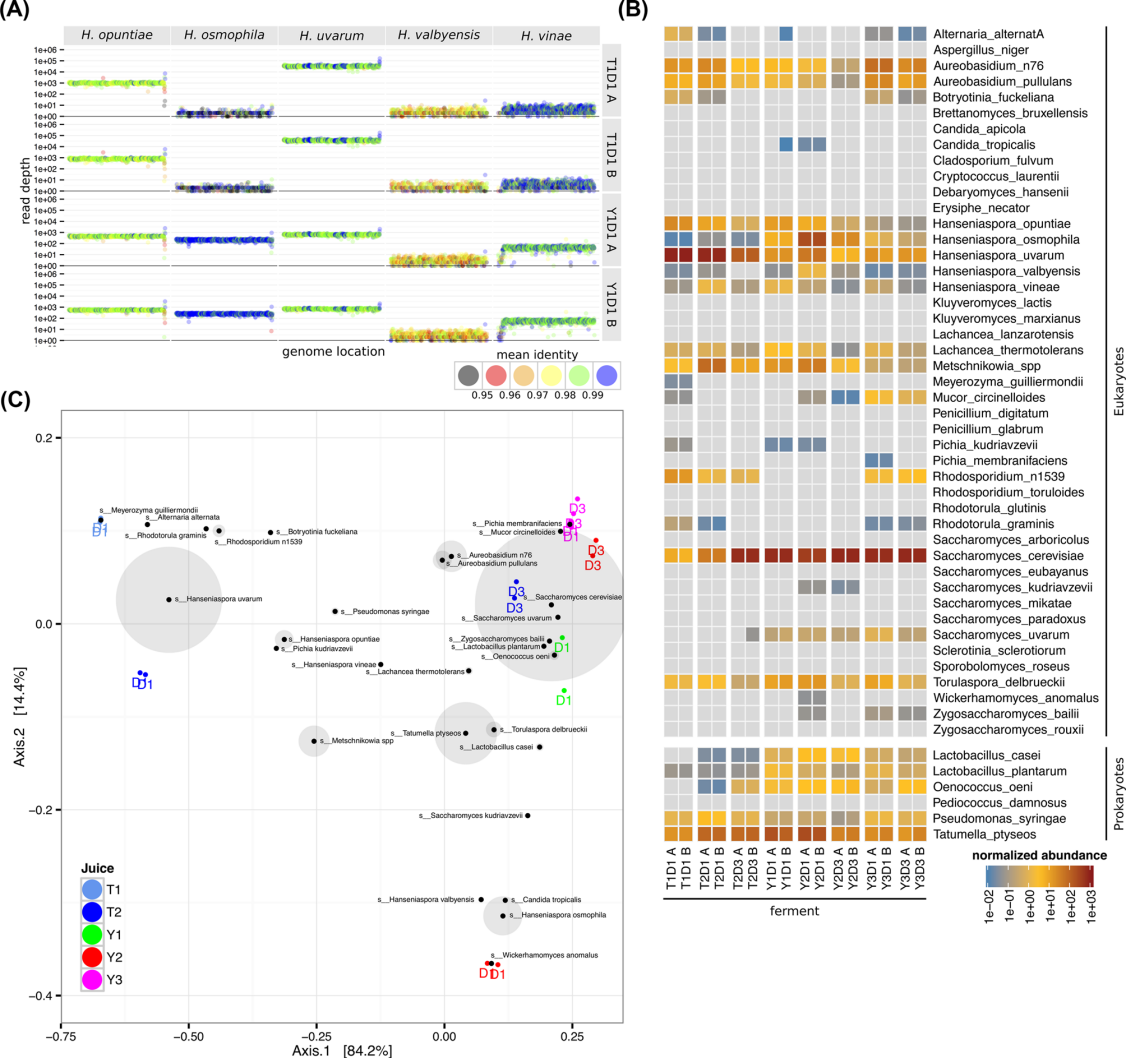
Shotgun metagenomic analysis of species. **(A)** Shotgun sequencing reads from each sample were mapped to the wine metagenome reference set. The total reads present in non-overlapping 10-kb windows across each genome were recorded relative to genomic location. In addition to total read number, the average identity of the reads in each window compared to the reference sequence was also calculated (id_factor). For clarity and space considerations, we depict here only the abundance measures for species within the *Hanseniaspora* genus for the 2 T2D1 and Y1D1 replicates (results for all samples are presented in Fig. S3). **(B)** Normalized average abundance values for each reference species in each sample. Values were normalized using total read numbers in each sample (including non-aligning reads), with final values represented per million reads in each 10-kb genomic window. **(C)** Bray-Curtis dissimilarity analysis of the shotgun abundance data. The weightings of each reference genome are overlaid on the plot, with the size of the gray circles around each node proportional to the total abundance of each reference genome across all of the samples (no shading for nodes >10).

For those reads that could be matched to the reference set, estimations of species abundance were made from average read coverage values from discrete 10-kb windows across each genome (See Fig. 2A for an example dataset for *Hanseniaspora spp.*; a full dataset is available in Fig. S3). In addition to read depth, the average identity between each read and the reference to which it mapped was also recorded. This provided an estimate of the evolutionary distance between each genomic reference and the strains or species present in each sample. These identity values were generally above 99% for the reference genomes, but were found to be significantly lower for reference sequences including *Mucor circinelloides*, *Pseudomonas syringae*, and *Hanseniaspora valbyensis*, suggesting that the actual species or strains present in the fermentation were significantly different than the reference used (Fig. S3).

To provide single abundance values for each reference genome in each sample, overall abundance measurements were derived from the average read depth of all 10-kb windows in each genomic sequence. An additional filter of at least 20% genome coverage was also applied to limit the effect of small numbers of windows with large coverage values, such as those derived from mis-mapping or potential small-scale horizontal transfer events from very high-abundance species against otherwise no- or low-abundance genomes (e.g., *S. cerevisiae* and *S. paradoxus*), from producing spurious abundance estimations. Using this technique, it was possible to detect the presence of 25 of the eukaryotic reference sequences and 5 prokaryotes across 5 orders of magnitude (Fig. 2B). As for the phylotyping, the major eukaryotic species that were identified include the *Aureobasidium spp.*, *Rhodotorula spp.*, and *Hanseniaspora spp.* early in the ferment progression and *Saccharomyces cerevisiae* late in fermentation.

Prokaryotic species, which could not be examined via the phylotyping experiments, included the malolactic wine bacterium *Oenococcus oeni* and various species of *Lactobacilli*, which are also commonly found in wine. Both *O. oeni* and the *Lactobacillus spp.* have been observed in previous microbiome projects in wine [10, 12, 14] and displayed expected increases in abundance during fermentation. In addition to the “wine-associated” bacteria, the plant pathogens *Pseudomonas syringae* and *Tatumella ptyseos*, which showed high levels of abundance early in fermentation, were also detected at high abundance; however, unlike the lactic acid bacteria, the abundance of these species declined as ferment progressed.

Comparing the shotgun metagenomic values obtained for the 4 control experiments to those expected from the estimated numbers of input cells showed that the outcomes of the shotgun analysis were within 2-fold of each other in all but 2 cases and that they were highly correlated with the ITS results (R^2^ = 0.99) (Table 2).

When multidimensional analysis (principal coordinate analysis [PCoA], using Bray-Curtis distances) was used to compare the shotgun samples, the presence of high amounts of *Hanseniaspora spp.* in 3 D1 samples (T1 D1, T2 D1, and Y2 D1) largely differentiated them from the 2 remaining samples. Within the 3 D1 samples that contained high levels of *Hanseniaspora spp.*, the D1 T1 and D1 T2 samples were primarily populated by *Hanseniaspora uvarum* while the Y2 sample contained roughly equal proportions of *Hanseniaspora uvarum* and *Hanseniaspora osmophila* (Fig. 2C).

### Comparison of shotgun and ITS-amplicon data

**Figure 3:**
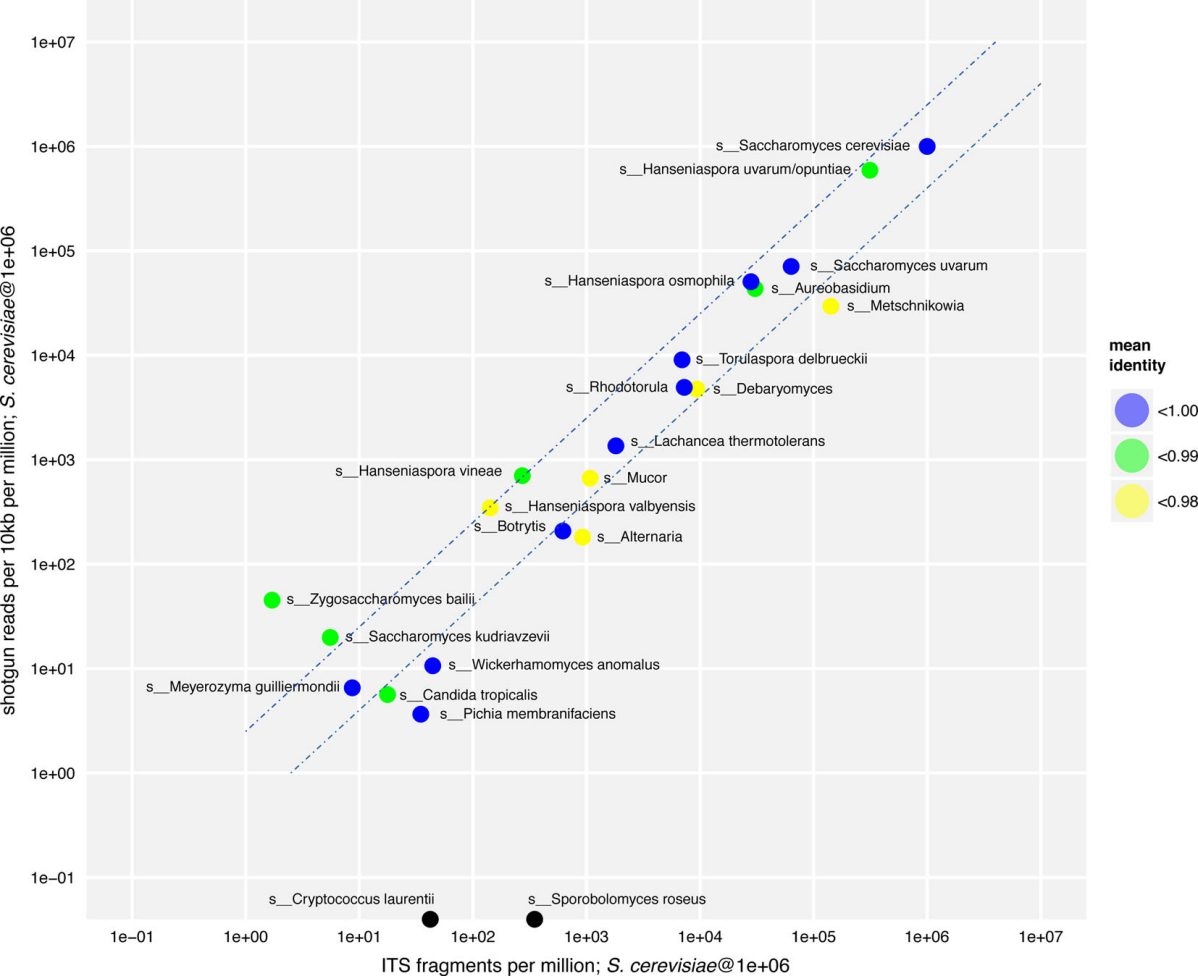
Comparison of ITS and shotgun abundance measurements. Normalized abundance measurements were scaled for both the shotgun and ITS experimental designs relative to a theoretical abundance of *S. cerevisiae* of 100% (1 million reads per 1 million ITS fragments or 1 million shotgun reads per 1 million reads per 10-kb genomic window). Dashed lines represent 2-fold variation between samples. The mean identity of the shotgun data relative to the reference genome used is also shown.

In addition to comparing the control values, it was possible to extract values for comparison from the full shotgun and ITS-amplicon datasets by comparing the results from a total of 23 comparable taxonomic identifiers that were present in both experimental types (Fig. 3). For most of these taxonomic identifiers, the normalized abundance values recorded from the shotgun and ITS-amplicon experiments were highly correlated (R^2^ = 0.93) and differed by 2-fold or less across a dynamic range of more than 4 orders of magnitude, with accuracy diminishing at levels below 100 reads/fragments per million. For those high-abundance species that were not within a 5-fold range, the previously identified ITS-amplicon overestimation bias for *Metschnikowia spp.* was recapitulated, confirming that this is a bias inherent in ITS analysis for this species.

### Potential implications

Uninoculated wine ferments represent a complex and dynamic microbial community. Metagenomics and phylotyping are now allowing for the detailed analysis of large numbers of fermentation samples, shining a light on the composition of these microbial mixtures. While the ITS phylotyping provides an accurate, high-throughput means to determine species abundance, the shotgun metagenomics uncovered at least 1 major example of amplicon bias, with the *Metschnikowia spp.* displaying a 10-fold over-representation. However, once biases such as these have been identified, they can be corrected in future ITS phylotyping datasets to provide a more accurate species representation. As more shotgun metagenomic and single-strain *de novo* assemblies for key wine species become available, the accuracy of both ITS-amplicon and shotgun studies will greatly increase. This will provide a key methodology for deciphering the influence of the microbial community on the wine flavor and aroma and how winemaking interventions may be used to shape these outcomes.

## Methods

### Mock control populations

To assemble control samples of known microbial composition, individual cultures of 7 wine yeasts (Table 1) were grown to stationary phase in YPD, with each culture microscopically enumerated using a hemocytometer. Specific numbers of cells from each culture were then mixed to produce 2 different control populations with proportions of individual wine yeasts that spanned several orders of magnitude (Table 1). Cell mixtures were then pelleted and DNA prepared for either phylotyping or shotgun abundance measurements as for the laboratory ferments.

### Laboratory ferments

For each of the laboratory-scale wild ferments, 20 l of Chardonnay grape juice was obtained directly from winery fermentation tanks immediately after crushing. Each 20 l sample was then split into 3 separate 3 l glass fermenters fitted with air-locks and fermented at 20°C with daily stirring; 50 ml samples were taken at D0, just after crush (11–12 Bé); D1, the onset of fermentation (10–11 Bé, ∼90% residual sugar); D2, mid-ferment (5–6 Bé, ∼50% residual sugar); D3, nearing the end of ferment (<3 Bé, <25% residual sugar). All ferments proceeded to dryness (0 Bé, <5 g/l residual sugar).

### DNA preparation

For each sample, 50 ml of fermenting juice was centrifuged for 10 minutes at 10 000 g, washed in 20 ml PBS, re-centrifuged, and then frozen at –80°C until processed. Total DNA was extracted from washed must pellets using the PowerFood Microbial DNA Isolation Kit (Mobio).

### ITS-amplicon preparation and analysis

Analysis of ITS abundance from ferment samples was performed using 2-step PCR amplification followed by next-generation amplicon sequencing (Fig. S1). First-round amplification of the ITS region was performed using the fungal-specific primers BITS (ACCTGCGGARGGATCA) and B58S3 (GAGATCCRTTGYTRAAAGTT) [9], which were modified to include both an inline barcode and Illumina adaptor sequences BITS-F1-N*xxx* and BITS-R1-N*xxx* (Table 4). One nanogram of DNA was used in each first-round PCR (20–30 cycles, 55°C annealing, 30-second extension, KAPA 2G Robust polymerase). Second-round amplification was performed using the Illumina adaptor sequence present in the first-round primers as an amplification target, with the remaining sequences required for dual-indexed sequencing on the Miseq platform added via overhang PCR (Fig. S1). For each sample, 2 ul of first-round PCR product was used (15 cycles, 55°C annealing, 30-second extension, KAPA 2G Robust polymerase). Following PCR, all samples were mixed into a single batch and column purified (minElute, Qiagen). ITS-amplicon pools were sequenced on the Illumina Miseq sequencing platform using 2 × 300 bp paired-end chemistry (Ramaciotti Centre for Functional Genomics, Randwick, Australia).

**Table 4:** ITS amplification primers used in this study

Primer	Sequence (Illumina adaptor^a^ | in-line barcode^b^ | spacer^c^ | ITS primer sequence^d^)
BITS-F1-N701	CCTACACGACGCTCTTCCGATCT|TAAGGCGA||ACCTGCGGARGGATCA
BITS-F1-N702	CCTACACGACGCTCTTCCGATCT|CGTACTAG|C|ACCTGCGGARGGATCA
BITS-F1-N703	CCTACACGACGCTCTTCCGATCT|AGGCAGAA|TC|ACCTGCGGARGGATCA
BITS-F1-N704	CCTACACGACGCTCTTCCGATCT|TCCTGAGC|ATC|ACCTGCGGARGGATCA
BITS-R1-N701	GTGACTGGAGTTCAGACGTGTGCTCTTCCGATCT|TAAGGCGA||GAGATCCRTTGYTRAAAGTT
BITS-R1-N702	GTGACTGGAGTTCAGACGTGTGCTCTTCCGATCT|CGTACTAG|C|GAGATCCRTTGYTRAAAGTT
BITS-R1-N703	GTGACTGGAGTTCAGACGTGTGCTCTTCCGATCT|AGGCAGAA|TC|GAGATCCRTTGYTRAAAGTT
BITS-R1-N704	GTGACTGGAGTTCAGACGTGTGCTCTTCCGATCT|TCCTGAGC|ATC|GAGATCCRTTGYTRAAAGTT

^a^Common sequence allowing for clustering on the Illumina flow cell.

^b^8-bp variable barcode.

^c^Variable length spacer (0–3 bp) used to unphase conserved amplicon regions and provide higher-quality sequencing on the Illumina platform.

^d^Sequences derived from [9].

Following sequencing, raw sequence data were quality trimmed (Trimmomatic v. 0.22 [27]; TRAILING:20 MINLEN:50) and adaptor trimmed at the 3^΄^ end to remove ITS adaptor sequences (cutadapt 1.2.1) [28], and the individual read pairs were overlapped to form single synthetic reads (FLASH 1.2.11 [29]; –max-overlap 1000 –allow-outies). These synthetic reads were then trimmed at both the 5^΄^ and 3^΄^ ends to remove any remaining Illumina adaptors that were directly adjacent to the inline barcodes (cutadapt 1.2.1 [28]; -a AGATCGGAAG -g CTTCCGATCT -e 0.1 -overlap 6) and sequentially partitioned according to the specific combination of inline barcode sequences at both the 5^΄^ and 3^΄^ end of each synthetic read using FASTX-Toolkit (v. 0.0.13; fastx_barcode_splitter.pl –bol –mismatches 1; [30]).

To calculate the abundance of individual amplicons, the entire dataset for all of the samples was dereplicated with USEARCH (v. 7.0.1990; -derep_full length, -size-out) [18] and each dereplicated OTU renamed according to the md5 checksum of the OTU sequence to provide unambiguous comparison of identical OTUs across experiments. Dereplicated OTUs were then clustered using SWARM (-z–differences 1 –fastidious [19]), with a minimum final OTU size of 10 implemented using custom scripts.

Following clustering, the likely taxonomic identity of the representative sequence of each OTU was determined using the assign_taxonomy.py module of QIIME using a modified form of the standard QIIME UNITE database in which any unclassified or unidentified sequences were removed and each ITS region was trimmed to the extent of the BITS primers used for the original ITS amplification (-t sh_taxonomy_qiime_ver6_dynamic_s_10.09.2014.txt -r sh_refs_qiime_ver6_dynamic_s_10.09.2014.BIT.unclassified.unidentified.fasta -m uclust –uclust_similarity = 0.98 –uclust_max_accepts = 10 –uclust_min_consensus_fraction = 0.4) [20]. In addition to the edited UNITE database, OTU annotations were also performed with an augmented version of the database in which several wine-specific, manually curated reference sequences were added and 3 UNITE reference sequences that were found to have erroneous annotations were either edited or removed (Supplemental File 1).

Once the results were established for the full dataset, individual dereplicated OTUs from each sample were matched back to those of the full dataset using custom scripts to provide a directly comparable and standardized assignment of each individual experimental result within the overall dataset. Final results were assembled in QIIME tabular format using custom scripts (Table S2).

Multidimensional data analysis was performed with the R phyloseq package [31] using PCoA and Bray Curtis dissimilarity measures based upon the 30 most abundant OTUs across the samples.

### Shotgun metagenomics analysis

DNA from 4 control populations and 16 fermentation samples from 2 wineries were subjected to whole-genome metagenomic sequencing. Random sequencing libraries were prepared using the Truseq nano protocol (Illumina) with a ∼350 bp insert size. Sequencing libraries were then pooled and run across 3 lanes of Illumina Hiseq 2 × 100 bp chemistry (Ramaciotti Centre for Functional Genomics, Australia).

Following sequencing, raw sequence data were first filtered to limit contaminating grapevine sequences by aligning each set of sequences against the Pinot Noir grapevine genome (CAAP00000000.3) [32] using Bowtie2 v. 2.2.5 in unpaired mode [33]. In all cases, >1% of total reads were found to match the grapevine genome. All unaligned reads for which both reads in a pair failed to align to the grapevine genome were retained for further analysis.

To provide a reference sequence for read mapping, whole genome sequences were collected, where possible, from a combination of species comprising either known grape and wine microbiota (including bacteria) or other fungal species identified as being present in the fermentations analyzed in this study via ITS phylotyping (Table S3). This reference sequence was divided up into discrete windows of 10 kb using Bedtools2 (v. 2.24.0; makewindows -w 10 000) [34].

Each of the filtered shotgun datasets was then aligned to this reference set using Bowtie2 in paired-end mode, with unaligned reads saved for later analysis (–fr –maxins 1500 –no-disconcordant –no-unal –un-conc) [33]. The resultant .sam files were sorted and converted to .bam format and filtered for low-quality alignments using Samtools (v. 1.2; view -bS -q 10 | sort) [35]. For each .bam file, the total read coverage in each 10-kb reference window was calculated using Bedtools2 (v. 2.24.0; coverage -counts) [34], with the mean, median, and adjusted mean (retain mean if ≥20% of the windows in that species contained ≥1 read; otherwise mean value of 0 applied) calculated from the bed window values for each species in each sample using custom scripts. In addition to coverage values, the average identity of each mapped read was calculated for each window using custom scripts that counted the number of mismatches per read (Bowtie2 XM: tag for each read) compared to overall read length.

### *De novo* metagenomic assembly

For the assembly of uncultivated sequences that were unrepresented in early versions of the shotgun reference collection, reads that failed to align during the shotgun metagenomic analysis were *de novo* assembled using SPADES (v. 3.5.0; –sc –careful) [36]. The likely taxonomic source of each contig was estimated using BLASTX (ncbi_blast-2.2.31+; -task blastx-fast -outfmt “7 std sscinames” -max_target_seqs 20) against the non-redundant database (nr; date 14 February 2015) and extracting the taxonomic source of the best blast hit. Contigs were then partitioned according this taxonomic grouping at the genus level, with genera being manually combined where appropriate.

### Comparison of shotgun and ITS phylotyping data

To compare the shotgun and ITS datasets, normalized abundance measurements for common species from both the shotgun and ITS experiments were scaled relative to a theoretical abundance of *S. cerevisiae* set at 100% (1 million reads per 1 million ITS fragments or 1 million shotgun reads per 1 million reads per 10-kb genomic window). This provided 2 sets of directly comparable values for downstream plotting and analysis.

## Abbreviations

Bé: Baumé; ITS: ribosomal DNA internal transcribed spacer; OTUs: operational taxonomic units; PCoA: principal coordinate analysis; PCR: polymerase chain reaction.

## Supplementary Material

Reviewer-1-Report-(Original-Submission).pdf

Reviewer-2-Report-(Original-Submission).pdf

Supplemental material

## Availability of supporting data and materials

The datasets supporting the results of this article are available in the GigaDB repository [17]. All sequencing data, including ITS barcoding and shotgun metagenomic sequencing and the non-*Saccharomyces* yeast *de novo* genome assemblies, have been deposited in Genbank under the BioProject accession number PRJNA305659.

Figure S1. Two-step amplification of the ITS region as an Illumina-ready amplicon. First-round amplification uses BITS and B58S3 primers [9] fused to inline barcodes and adaptor sequences. Second-round amplification takes advantage of the common Illumina adaptors to add Illumina indexing sequences and the P7 and P5 adaptors that are required for flow cell adherence and amplification.

Figure S2. Correlation analysis of replicate ferments. Pair-wise comparisons were performed between triplicate samples from the 4 fermentation time points across the 5 different juices. Raw abundance measurements were compared for all significant OTUs (>10 reads). R^2^ values are presented for each pairwise comparison (Corr), with the density of data points indicated on the diagonal plots.

Figure S3. Shotgun metagenomic analysis of species abundance in wild fermentation via read mapping. Shotgun sequencing reads from each sample were mapped to the wine metagenome reference set. The total reads present in non-overlapping 10-kb windows across each genome were recorded relative to genomic location. In addition to total read number, the average identity of the reads in each window compared to the reference sequence was also calculated (id_factor).

S1 Table. Samples analyzed by ITS phylotyping (meta-barcoding). All samples studied by ITS phylotyping are listed, including grape variety, location, and total number of reads processed.

S2 Table. ITS phylotyping abundance estimations. The results of OTU clustering are provided for all samples studied by ITS phylotyping. The results of automated taxonomy assignment (QIIME) and manual BLAST annotation are also shown, along with the final taxonomic classification used for downstream analysis (kingdom, phylum, class, order, family, genus, and species).

S3 Table. Wine reference genomes. A full list of genome sequences (and their sources) used in the wine reference consortium.

S4 Table. MetaPhlAn analysis of unaligned metagenomic reads. MetaPhLan results for the collection of unaligned shotgun metagenomic reads.

S1 File. Customized UNITE ITS database. The custom processed UNITE ITS database used for automated QIIME taxonomy assignment

## References

[1] Fleet GH Wine yeasts for the future. FEMS Yeast Res 2008;8:979–95. 18793201 10.1111/j.1567-1364.2008.00427.x

[2] Beltran G, Torija MJ, Novo M Analysis of yeast populations during alcoholic fermentation: a six year follow-up study. Syst Appl Microbiol 2002;25:287–93. 12353885 10.1078/0723-2020-00097

[3] Combina M, Elía A, Mercado L Dynamics of indigenous yeast populations during spontaneous fermentation of wines from Mendoza, Argentina. Int J Food Microbiol 2005;99:237–43. 15808358 10.1016/j.ijfoodmicro.2004.08.017

[4] Fleet GH Growth of yeasts during wine fermentations. J Wine Res 1990;1:211–23.

[5] Fleet GH, Lafon-Lafourcade S, Ribéreau-Gayon P Evolution of yeasts and lactic acid bacteria during fermentation and storage of Bordeaux wines. Appl Environ Microbiol 1984;48:1034–8. 16346661 10.1128/aem.48.5.1034-1038.1984 PMC241671

[6] Martini A, Ciani M, Scorzetti G Direct enumeration and isolation of wine yeasts from grape surfaces. Am J Enol Vitic 1996;47:435–40.

[7] Mortimer R, Polsinelli M On the origins of wine yeast. Res Microbiol 1999;150:199–204. 10229949 10.1016/s0923-2508(99)80036-9

[8] Caporaso JG, Lauber CL, Walters WA Global patterns of 16S rRNA diversity at a depth of millions of sequences per sample. Proc Natl Acad Sci U S A 2011;108(suppl 1):4516–22. 20534432 10.1073/pnas.1000080107 PMC3063599

[9] Bokulich NA, Mills DA Improved selection of internal transcribed spacer-specific primers enables quantitative, ultra-high-throughput profiling of fungal communities. Appl Environ Microbiol 2013;79:2519–26. 23377949 10.1128/AEM.03870-12 PMC3623200

[10] Bokulich NA, Joseph CML, Allen G Next-generation sequencing reveals significant bacterial diversity of botrytized wine. PLoS One 2012;7:e36357. 22563494 10.1371/journal.pone.0036357 PMC3341366

[11] Bokulich NA, Thorngate JH, Richardson PM Microbial biogeography of wine grapes is conditioned by cultivar, vintage, and climate. Proc Natl Acad Sci U S A 2014;111:E139–48. 24277822 10.1073/pnas.1317377110 PMC3890796

[12] Pinto C, Pinho D, Cardoso R Wine fermentation microbiome: a landscape from different Portuguese wine appellations. Front Microbiol 2015;6:905. 26388852 10.3389/fmicb.2015.00905 PMC4555975

[13] Taylor MW, Tsai P, Anfang N Pyrosequencing reveals regional differences in fruit-associated fungal communities. Environ Microbiol 2014;16:2848–58. 24650123 10.1111/1462-2920.12456 PMC4257574

[14] Bokulich NA, Collins TS, Masarweh C Associations among wine grape microbiome, metabolome, and fermentation behavior suggest microbial contribution to regional wine characteristics. mBio 2016;7:e00631–16. 27302757 10.1128/mBio.00631-16 PMC4959672

[15] Pinto AJ, Raskin L PCR biases distort bacterial and archaeal community structure in pyrosequencing datasets. PLoS One 2012;7:e43093. 22905208 10.1371/journal.pone.0043093 PMC3419673

[16] Sharpton TJ An introduction to the analysis of shotgun metagenomic data. Front Plant Sci 2014;5:209. 24982662 10.3389/fpls.2014.00209 PMC4059276

[17] Sternes PR, Lee D, Kutyna DR Supporting data for “A combined meta-barcoding and shotgun metagenomic analysis of spontaneous wine fermentation”. GigaScience Database 2017 http://dx.doi.org/10.5524/100309. 10.1093/gigascience/gix040 PMC5570097 28595314

[18] Edgar RC Search and clustering orders of magnitude faster than BLAST. Bioinformatics 2010;26:2460–1. 20709691 10.1093/bioinformatics/btq461

[19] Mahé F, Rognes T, Quince C Swarm: robust and fast clustering method for amplicon-based studies. Peer J 2014;2:e593. 25276506 10.7717/peerj.593 PMC4178461

[20] Caporaso JG, Kuczynski J, Stombaugh J QIIME allows analysis of high-throughput community sequencing data. Nat Methods 2010;7:335–6. 20383131 10.1038/nmeth.f.303 PMC3156573

[21] Cordero-Bueso G, Arroyo T, Serrano A Influence of the farming system and vine variety on yeast communities associated with grape berries. Int J Food Microbiol 2011;145:132–9. 21185102 10.1016/j.ijfoodmicro.2010.11.040

[22] Cordero-Bueso G, Arroyo T, Valero E A long term field study of the effect of fungicides penconazole and sulfur on yeasts in the vineyard. Int J Food Microbiol 2014;189:189–94. 25171112 10.1016/j.ijfoodmicro.2014.08.013

[23] Martins G, Vallance J, Mercier A Influence of the farming system on the epiphytic yeasts and yeast-like fungi colonizing grape berries during the ripening process. Int J Food Microbiol 2014;177:21–8. 24603471 10.1016/j.ijfoodmicro.2014.02.002

[24] Setati ME, Jacobson D, Andong U-C The vineyard yeast microbiome, a mixed model microbial map. PLoS One 2012;7:e52609. 23300721 10.1371/journal.pone.0052609 PMC3530458

[25] Segata N, Waldron L, Ballarini A Metagenomic microbial community profiling using unique clade-specific marker genes. Nat Methods 2012;9:811–4. 22688413 10.1038/nmeth.2066 PMC3443552

[26] Martins G, Lauga B, Miot-Sertier C Characterization of epiphytic bacterial communities from grapes, leaves, bark and soil of grapevine plants grown, and their relations. PLoS One 2013;8:e73013. 24023666 10.1371/journal.pone.0073013 PMC3758280

[27] Bolger AM, Lohse M, Usadel B Trimmomatic: a flexible trimmer for Illumina sequence data. Bioinformatics 2014;30:2114–20. 24695404 10.1093/bioinformatics/btu170 PMC4103590

[28] Martin M Cutadapt removes adapter sequences from high-throughput sequencing reads. EMBnet J 2011;17:10.

[29] Magoč T, Salzberg SL FLASH: fast length adjustment of short reads to improve genome assemblies. Bioinformatics 2011;27:2957–63. 21903629 10.1093/bioinformatics/btr507 PMC3198573

[30] http://hannonlab.cshl.edu/fastx_toolkit/.

[31] McMurdie PJ, Holmes S phyloseq: an R package for reproducible interactive analysis and graphics of microbiome census data. PLoS One 2013;8:e61217. 23630581 10.1371/journal.pone.0061217 PMC3632530

[32] Jaillon O, Aury J-M, Noel B The grapevine genome sequence suggests ancestral hexaploidization in major angiosperm phyla. Nature 2007;449:463–7. 17721507 10.1038/nature06148

[33] Langmead B, Salzberg SL Fast gapped-read alignment with Bowtie 2. Nat Methods 2012;9:357–9. 22388286 10.1038/nmeth.1923 PMC3322381

[34] Quinlan AR, Hall IM BEDTools: a flexible suite of utilities for comparing genomic features. Bioinformatics 2010;26:841–2. 20110278 10.1093/bioinformatics/btq033 PMC2832824

[35] Li H, Handsaker B, Wysoker A The Sequence Alignment/Map format and SAMtools. Bioinformatics 2009;25:2078–9. 19505943 10.1093/bioinformatics/btp352 PMC2723002

[36] Bankevich A, Nurk S, Antipov D SPAdes: a new genome assembly algorithm and its applications to single-cell sequencing. J Comput Biol 2012;19:455–77. 22506599 10.1089/cmb.2012.0021 PMC3342519

