# Supplementary figures and images for: A combined meta-barcoding and shotgun metagenomic analysis of spontaneous wine fermentation
Source: Gigascience. 2017 Jun 8;6(7):1–10. doi: 10.1093/gigascience/gix040 (PMC5570097; doi:10.1093/gigascience/gix040)

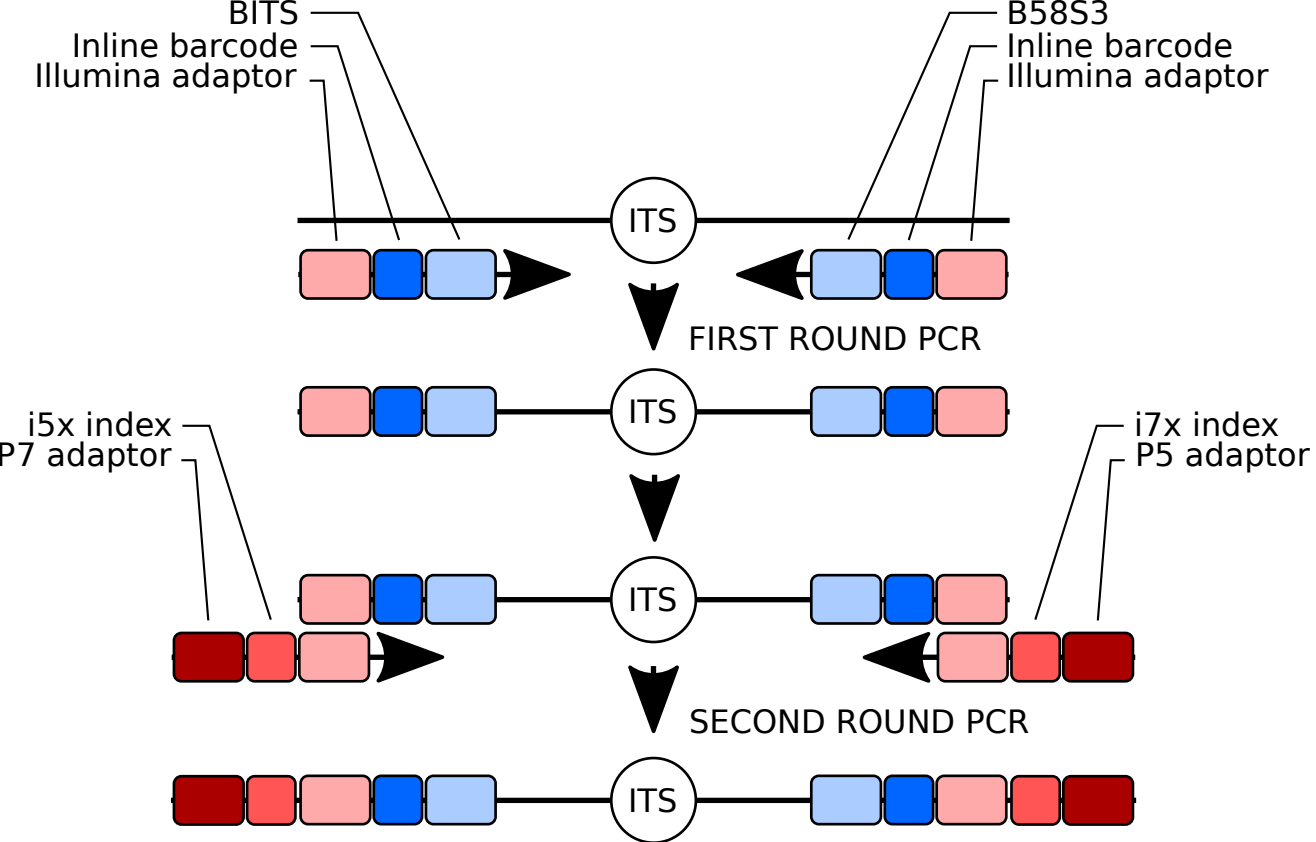

Supplement: Supplemental material [file gix040_Supp.zip › Sternes_etal_FigS1.pdf]

control

1

2

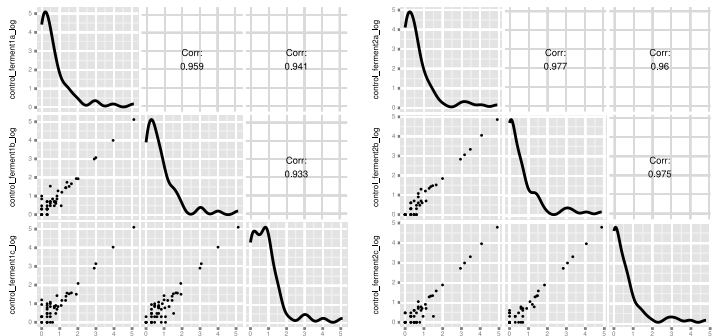

T1

D0

D1

D2

D3

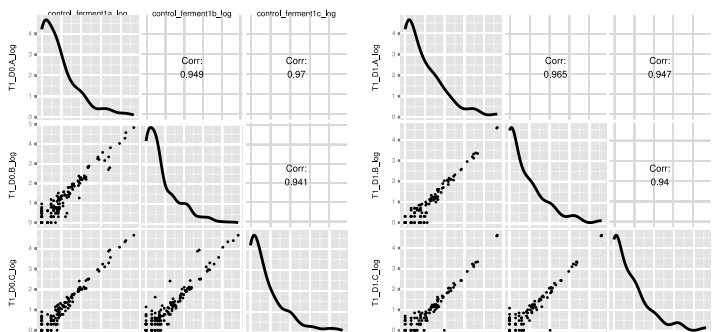

T2

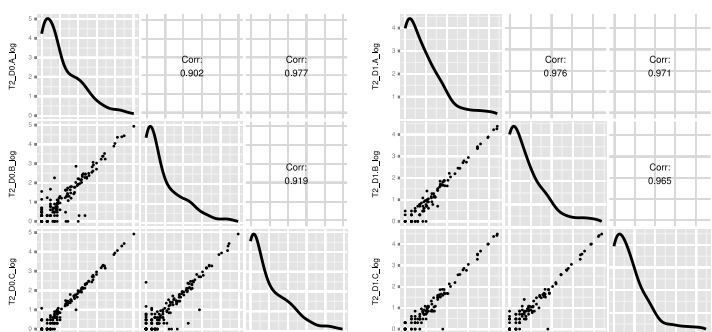

Y1

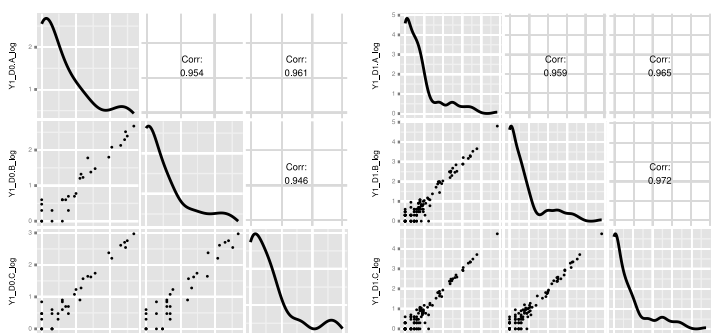

Y2

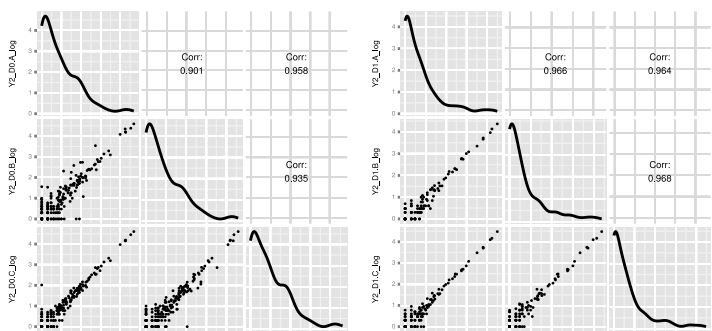

Y3

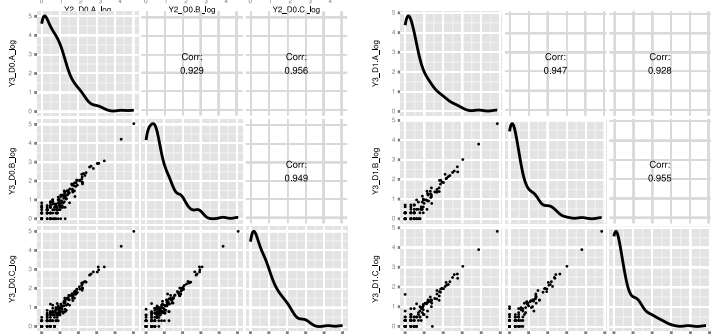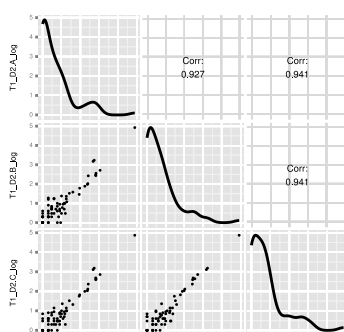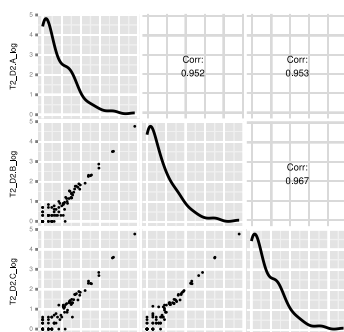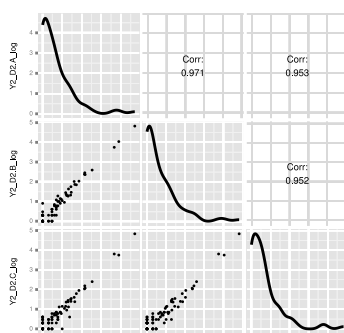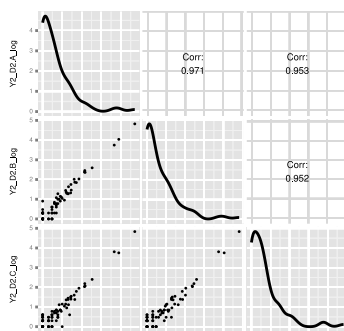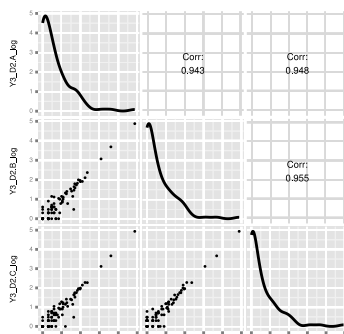

Supplement: Supplemental material [file gix040_Supp.zip › Sternes_etal_FigS2.pdf]

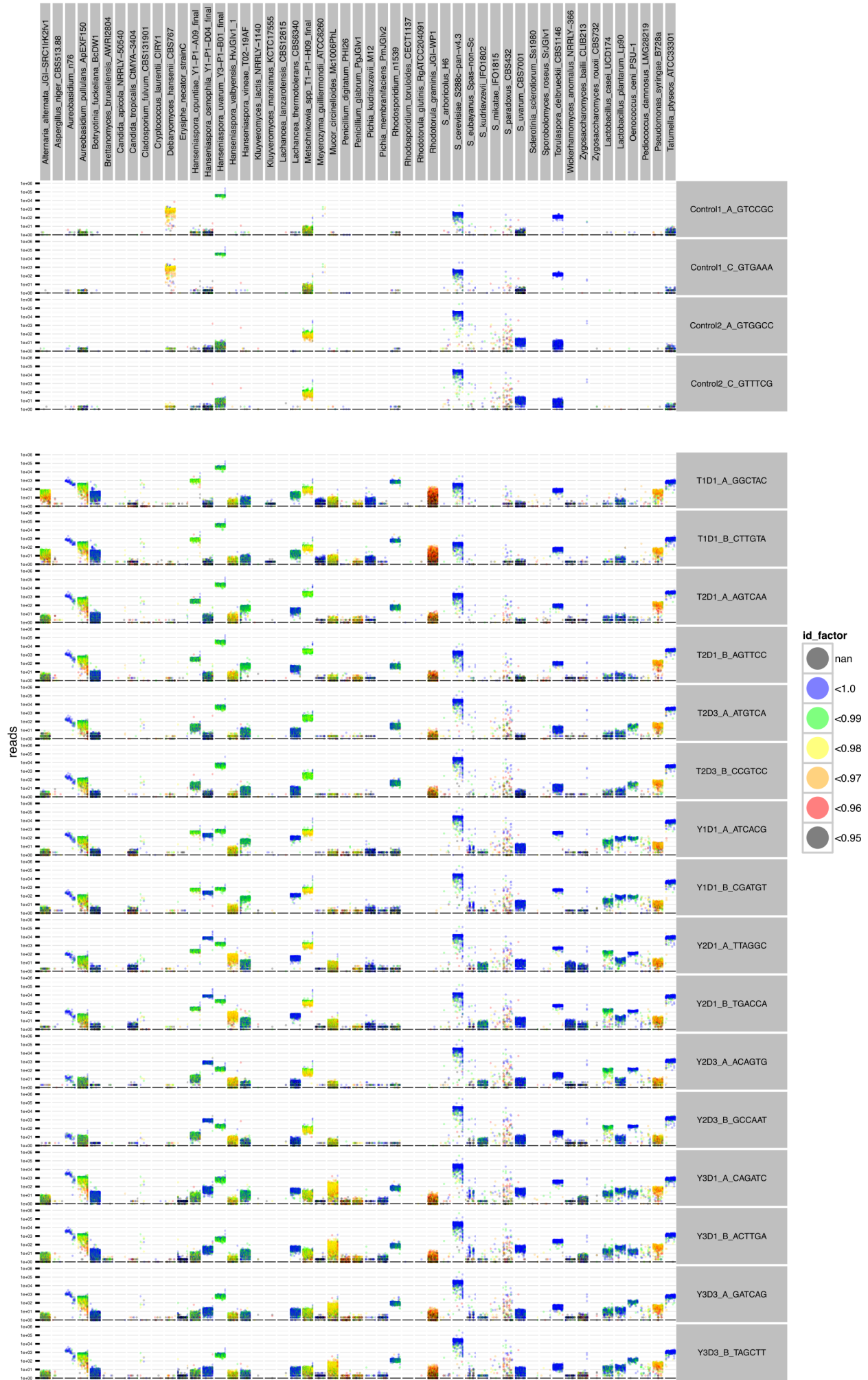

Supplement: Supplemental material [file gix040_Supp.zip › Sternes_etal_FigS3.pdf]
