# Supplementary material for: A combined meta-barcoding and shotgun metagenomic analysis of spontaneous wine fermentation
Source: Gigascience. 2017 Jun 8;6(7):1–10. doi: 10.1093/gigascience/gix040 (PMC5570097; doi:10.1093/gigascience/gix040)
Supplement: Reviewer-1-Report-(Original-Submission).pdf [file gix040_Reviewer-1-Report-(Original-Submission).pdf]

Reviewer Report

Title: Sequencing, de novo assembling and annotating the genome of the endangered Chinese crocodile lizard Shinisaurus crocodilurus

Version: Revision 1 **Date:** 03/17/2017

Reviewer name: Marc Tollis

Reviewer Comments to Author:

The authors present a genome assembly for a taxonomically distinct and CITES-listed anguimporh lizard, Shinisaurus. They generated 149X sequence coverage and trimmed the raw data to provide 70X coverage. The authors use a kmer depth analysis to estimate the genome size at 1.95Gb Using a combination of short- and long-insert libraries, the authors assemble a 2.24 Gb genome with a scaffold N50 of ~1.5Mb and contig N50 of ~11.7kb. Estimated gene content based on BUSCO analysis predicted ~15% of vertebrate genes to be missing from the assembly. Various repeat annotation methods were employed with differing results, however the de novo repeat modeling methods predicts the most repetitive content at ~59%. They use homology-based and abi initio gene prediction methods as well as functional assignment to annotate 20,010 protein coding genes in the assembly which meets expectations for a vertebrate. In total, the authors use technically sound methods to produce an assembly with impressive scaffold contiguity and a large number of genes. The assembly should be useful in any future population genomic efforts for species conservation and provides a much-needed anguimorph squamate genome which will be useful for reptilian comparative genomics. The manuscript is written in clear English and the methods are outlined well enough for reproducibility. I suggest rapid publication provided my brief comments can be addressed:

One comment I have stems from line # 57 in the manuscript: "This lizard is still alive in the collection of Ocean Park Hong Kong when the manuscript is finished". This is a little confusing, do you mean you are confirming the animal was still alive as of a certain date? This sentence should be edited or removed.

The analysis of the repeat annotation output is very brief, precisely which LINEs are comprising 10.2% of the genome? It would be interesting to know if this anguimorph genome in terms of repeat content is more similar to known iguanians (i.e. Anolis and Pogona) or snakes (i.e. Python and Ophiophagus). A very simple description could be used to suggest a potential phylogenetic reconciliation of relationships between iguanians, snakes, and anguimorphs which is an ongoing debate in the literature.

Concerning your Table 4: BUSCO results are highly contingent on either certain variables set in the OS environment or dependency software versions, sometimes producing vastly inconsistent results. You may not choose not to do this, but I would highly recommend re-running the analyses on another platform just to ensure the accuracy of your gene predictions.

Level of Interest

Please indicate how interesting you found the manuscript: An article whose findings are important to those with closely related research interests

Quality of Written English

Please indicate the quality of language in the manuscript: Acceptable

Declaration of Competing Interests

Please complete a declaration of competing interests, considering the following questions:

- Have you in the past five years received reimbursements, fees, funding, or salary from an organisation that may in any way gain or lose financially from the publication of this manuscript, either now or in the future?
- Do you hold any stocks or shares in an organisation that may in any way gain or lose financially from the publication of this manuscript, either now or in the future?
- Do you hold or are you currently applying for any patents relating to the content of the manuscript?
- Have you received reimbursements, fees, funding, or salary from an organization that holds or has applied for patents relating to the content of the manuscript?
- Do you have any other financial competing interests?
- Do you have any non-financial competing interests in relation to this paper?

If you can answer no to all of the above, write 'I declare that I have no competing interests' below. If your reply is yes to any, please give details below.

I declare that I have no competing interests.

I agree to the open peer review policy of the journal. I understand that my name will be included on my report to the authors and, if the manuscript is accepted for publication, my named report including any attachments I upload will be posted on the website along with the authors' responses. I agree for my report to be made available under an Open Access Creative Commons CC-BY license (http://creativecommons.org/licenses/by/4.0/). I understand that any comments which I do not wish to be included in my named report can be included as confidential comments to the editors, which will not be published.

I agree to the open peer review policy of the journal
