# Supplementary material for: A combined meta-barcoding and shotgun metagenomic analysis of spontaneous wine fermentation
Source: Gigascience. 2017 Jun 8;6(7):1–10. doi: 10.1093/gigascience/gix040 (PMC5570097; doi:10.1093/gigascience/gix040)
Supplement: Supplemental material [file gix040_Supp.zip › Figure Table Legends.doc]

**Figure S1**. Two step amplification of the ITS region as an Illumina-ready amplicon. First round amplification uses BITS and B58S3 primers [9] fused to inline barcodes and adaptor sequences. Second round amplification takes advantage of the common Illumina adaptors to add Illumina indexing sequences and the P7 and P5 adaptors that are required for flow cell adherence and amplification.

**Figure S2.** Correlation analysis of replicate ferments. Pair wise comparisons were performed between triplicate samples from the four fermentation time points across the five different juices. Raw abundance measurements were compared for all significant OTUs (>10 reads). R^2^ values are presented for each pairwise comparison (Corr), with the density of data points indicated on the diagonal plots.

**Figure S3.** Shotgun metagenomic analysis of species abundance in wild fermentation via read mapping. Shotgun sequencing reads from each sample were mapped to the wine metagenome reference set. The total reads present in non-overlapping 10 kb windows across each genome were recorded relative to genomic location. In addition to total read number, the average identity of the reads in each window compared to the reference sequence was also calculated (id_factor).

**S1 Table. Samples analyzed by ITS phylotyping (meta-barcoding).** All samples studied by ITS phylotyping are listed, including grape variety, location and total number of reads processed.

**S2 Table. ITS-phylotyping abundance estimations.** The results of OTU clustering are provided for all samples studied by ITS phylotyping. The results of automated taxonomy assignment (QIIME) and manual BLAST annotation are also shown along with the final taxonomic classification used for downstream analysis (kingdom, phylum, class, order, family, genus and species)

**S3 Table. Wine reference genomes.** A full list of genome sequences (and their sources) used in wine reference consortium.

**S4 Table. MetaPhlAn analysis of unaligned metagenomic reads.** MetaPhLan results for the collection of unaligned shotgun metagenomic reads.

**S1 File. Customized UNITE ITS database.** The custom processed UNITE ITS database used for automated QIIME taxonomy assignment.
